# A radiographic study of the distal femoral epiphysis

**DOI:** 10.1007/s11832-015-0660-1

**Published:** 2015-06-05

**Authors:** Cynthia V. Nguyen, Janelle D. Greene, Daniel R. Cooperman, Raymond W. Liu

**Affiliations:** Division of Pediatric Orthopaedic Surgery, Rainbow Babies and Children’s Hospital, Case Western Reserve University, 11100 Euclid Avenue, RBC 6081, Cleveland, OH 44106 USA; Department of Orthopaedics and Rehabilitation, Yale University School of Medicine, P.O. Box 208071, New Haven, CT 06520-8071 USA

**Keywords:** Distal femur, Epiphysis, Physis, Radiographs

## Abstract

**Purpose:**

Previous studies have described the complex undulation pattern in the distal femoral physis. We investigated whether standard radiographs can visualize these landmarks, in order to guide hardware placement in the distal immature femur.

**Methods:**

We studied 36 cadaveric immature femora in specimens 3 to 18 years of age. Anteroposterior (AP) and lateral radiographs were obtained with and without flexible radiodense markers placed on the major undulations and were analyzed to determine the relative height or depth of each topographical landmark. Intraclass correlation coefficients (ICCs) were calculated between measurements taken with and without markers for each undulation on each view.

**Results:**

Examination of the specimens confirmed a central peak and anteromedial and posterolateral valleys as the major physeal structures. AP radiographs without markers correlated well with marked AP radiographs for all three landmarks (ICC = 0.92, 0.92, 0.91), but the lateral radiographs had lower correlations for the posterolateral valley (ICC = 0.36). The correlation between AP and lateral radiographs without markers on the posterolateral valley was also decreased compared to the other two landmarks (ICC = 0.28 versus 0.57 for the central ridge and 0.62 for the anteromedial valley).

**Conclusions:**

This is the first study to rigorously evaluate radiographic visibility of the distal femur physeal undulations. The position of the central ridge, anteromedial valley, and posterolateral valley are reliably seen on AP radiographs, while the lateral view is less consistent, especially for the posterolateral valley. We recommend that caution should be taken when placing screws near the posterolateral aspect of the epiphysis, as lateral views do not visualize those undulations well.

## Introduction

When operating near physes, surgeons must take great care not to inadvertently injure the growth plate. It has been known for quite some time that damage to the physis, particularly the periphery, could lead to potential bar formation and growth disturbance [1–5]. In addition, fractures in the distal femur lead to a particularly high rate of growth arrest, presumably secondary to the undulating nature of the distal femur physis [6]. An attempt at minimizing these complications requires a detailed understanding of pediatric bony anatomy, as well as the prudent use of imaging techniques. A previous direct anatomic study has described the complex undulation pattern of the distal femoral physis and identified three major landmarks relevant on the metaphyseal and epiphyseal sides: a central peak, an anteromedial valley, and a posterolateral valley [6].

Fluoroscopy is routinely used to guide hardware placement when operating near the physis, such as during operative fixation of a distal femur fracture, graft placement in a pediatric medial patellofemoral ligament (MPFL) reconstruction or an all epiphyseal or partial transphyseal anterior cruciate ligament (ACL) reconstruction [7–9]. Recently, the technique of plate hemiepiphysiodesis has increased the indications for the placement of screws in the distal femoral epiphysis [10, 11].

To our knowledge, the reliability of radiographic imaging in visualizing the undulations of the distal femoral physis has never been quantitatively evaluated. The purpose of this study was to investigate how well the complex topography of the distal femoral physis can be visualized with plain radiographs.

## Materials and methods

### Obtaining radiographs

We studied 36 cadaveric immature femora in specimens 3 to 18 years of age contained from the Hamann-Todd Osteological Collection at the Cleveland Museum of Natural History. Inclusion criteria were the presence of separate epiphyseal and metaphyseal pieces, reflecting an open growth plate that was removed during standard specimen processing. All specimens in the collection were considered, but only 36 were determined to be adequate for our study. Exclusion criteria were obvious signs of damage or deformity to the specimen.

**Fig. 1:**
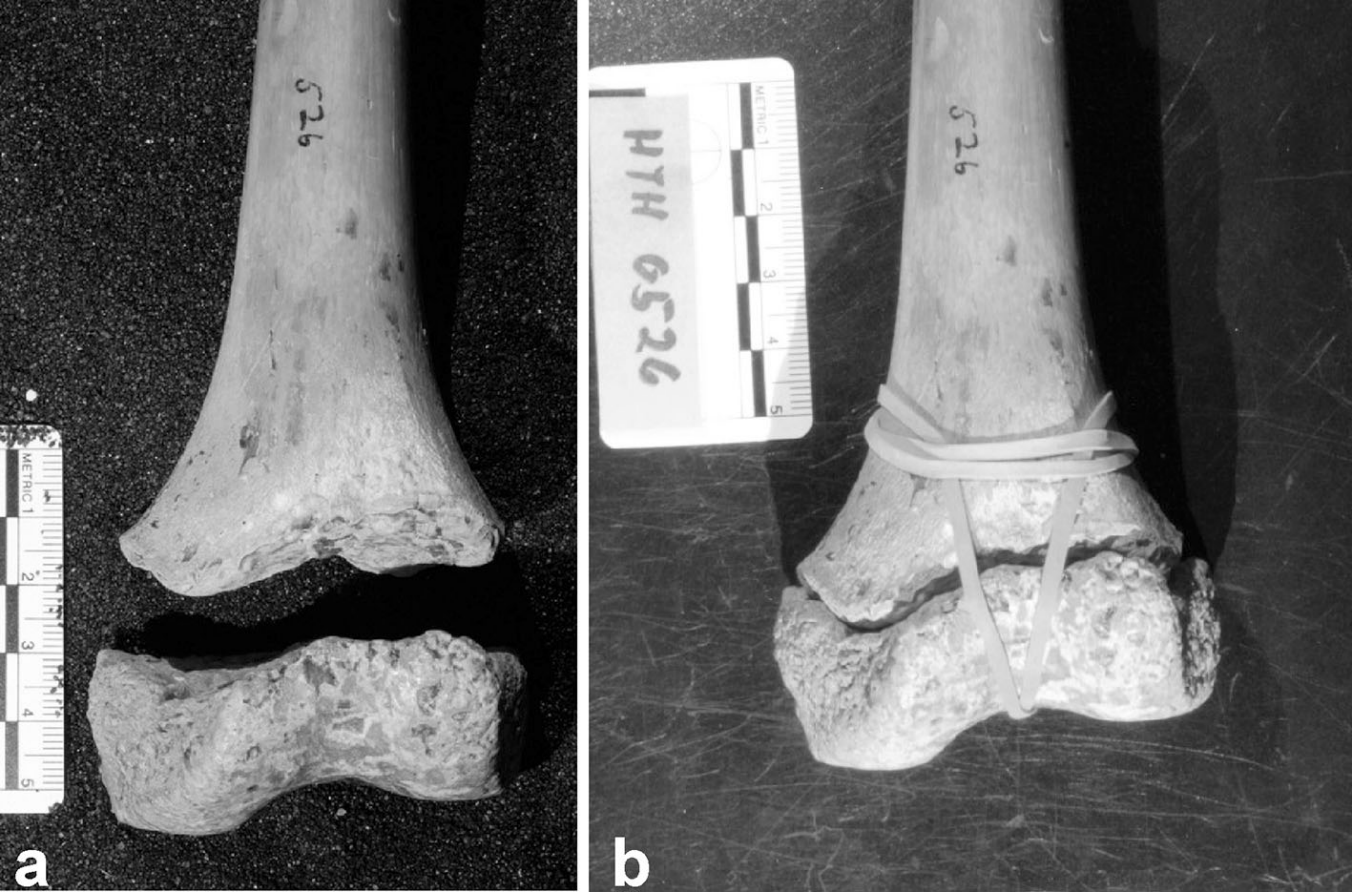
The lock and key relationship between epiphysis and metaphysis. Cadaveric distal femur of an 11-year-old female. **a** Separated epiphysis and metaphysis. **b** Distal femur reassembled

Each study specimen was reassembled into a complete distal femur unit by attaching the epiphyseal piece to the metaphyseal piece using rubber bands. The epiphyseal pieces fit into the corresponding distal metaphysis in a lock and key relationship via the interdigition of the physeal undulations (Fig. 1). Standard radiographs were taken of each distal femur unit in the anteroposterior (AP) and lateral views using a Hewlett Packard Faxitron model A3855A (Hewlett Packard, Palo Alto, CA). The specimens were carefully positioned using foam blocks to replicate standard positioning for radiographs of the distal femur so that, on the lateral view, the femoral condyles were superimposed and, on the AP view, the specimens were positioned with the posterior condyles and the proximal femurs all resting on the floor of the machine. A scale was also included in each radiograph as a standardized size reference.

The epiphyseal piece was then detached and small pieces of radiodense magnetic tape (Tree House Studio 3/4” Magnetic Tape, Oklahoma City, OK) were placed at the apices of the previously defined central peak, anteromedial valley, and anterolateral valley. Each distal femur unit was then reassembled and repeat AP and lateral radiographs were taken with the radiodense markers in place.

### Analyzing radiographs

The hard copy radiographs were converted into electronic files using a digital camera. A line was then drawn on each radiograph to represent the physeal plate. For the AP view radiographs, the lines were drawn based on the medial and lateral aspects of the metaphyseal-epiphyseal junctions. On the lateral view radiographs, the lines were drawn based on the anterior and posterior aspects of the metaphyseal-epiphyseal junctions.

**Fig. 2:**
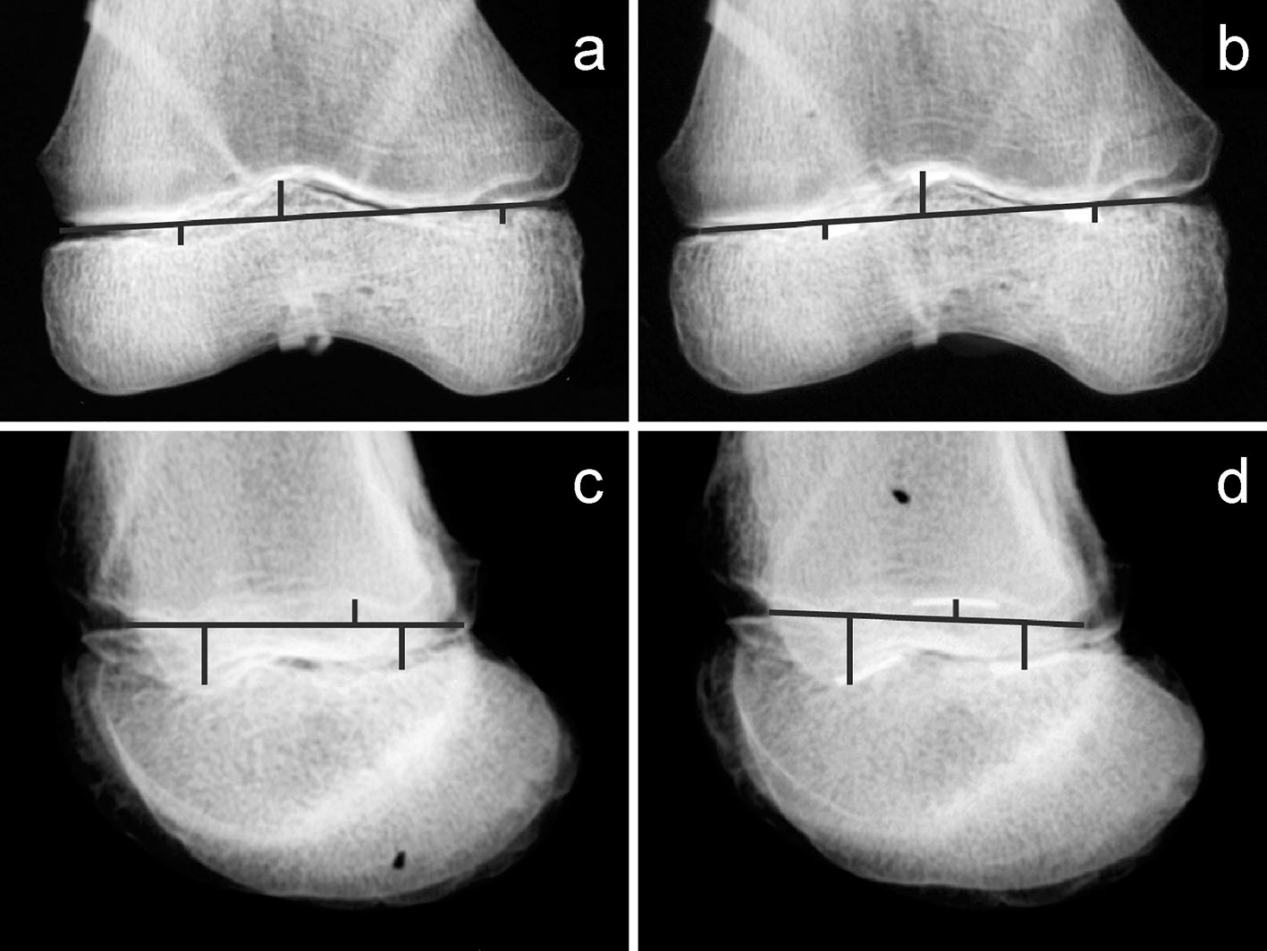
Example of radiographs taken from a cadaveric distal femur of an 8-year-old female. The lines are drawn to indicate the measurements. **a** Anteroposterior (AP) radiograph without markers. **b** AP radiograph with markers. **c** Lateral radiograph without markers. **d** Lateral radiograph with markers

On the radiographs taken of the distal femurs with radiodense markers, the location of the highest part of the peak and the lowest parts of the valleys were noted. The distances between the physeal line and the landmarks were measured. The physeal plate line and distances to the markers were placed and drawn onto the digital image by one author, and then each placement was reviewed by two other authors. Using a reference scale for calibration, the distances were converted to millimeters. After one week, the same process was repeated for the radiographs in which the landmarks were not marked (Fig. 2).

For each landmark, the measurements using all techniques were averaged and Pearson product moment correlation coefficients were calculated using Microsoft Excel (Microsoft, Redmond, WA) to compare their heights with increasing age. Intraclass correlation coefficients (ICCs) were calculated between measurements taken with and without markers for each undulation on each view, as well as between the views of each landmark using SPSS (IBM Corporation, Armonk, NY).

To establish interobserver reliability, 20 specimens were randomly chosen to be remeasured by another author. These specimens were also remeasured by the primary measuring author to establish intraobserver reliability. ICCs were calculated using the SPSS statistical package (IBM Corporation, Armonk, NY). Following established recommendations, we considered an ICC of <0.4 to be poor, 0.4–0.75 to be fair to good, and >0.75 to be excellent [12, 13].

No human or animal rights were violated during this research.

## Results

**Fig. 3:**
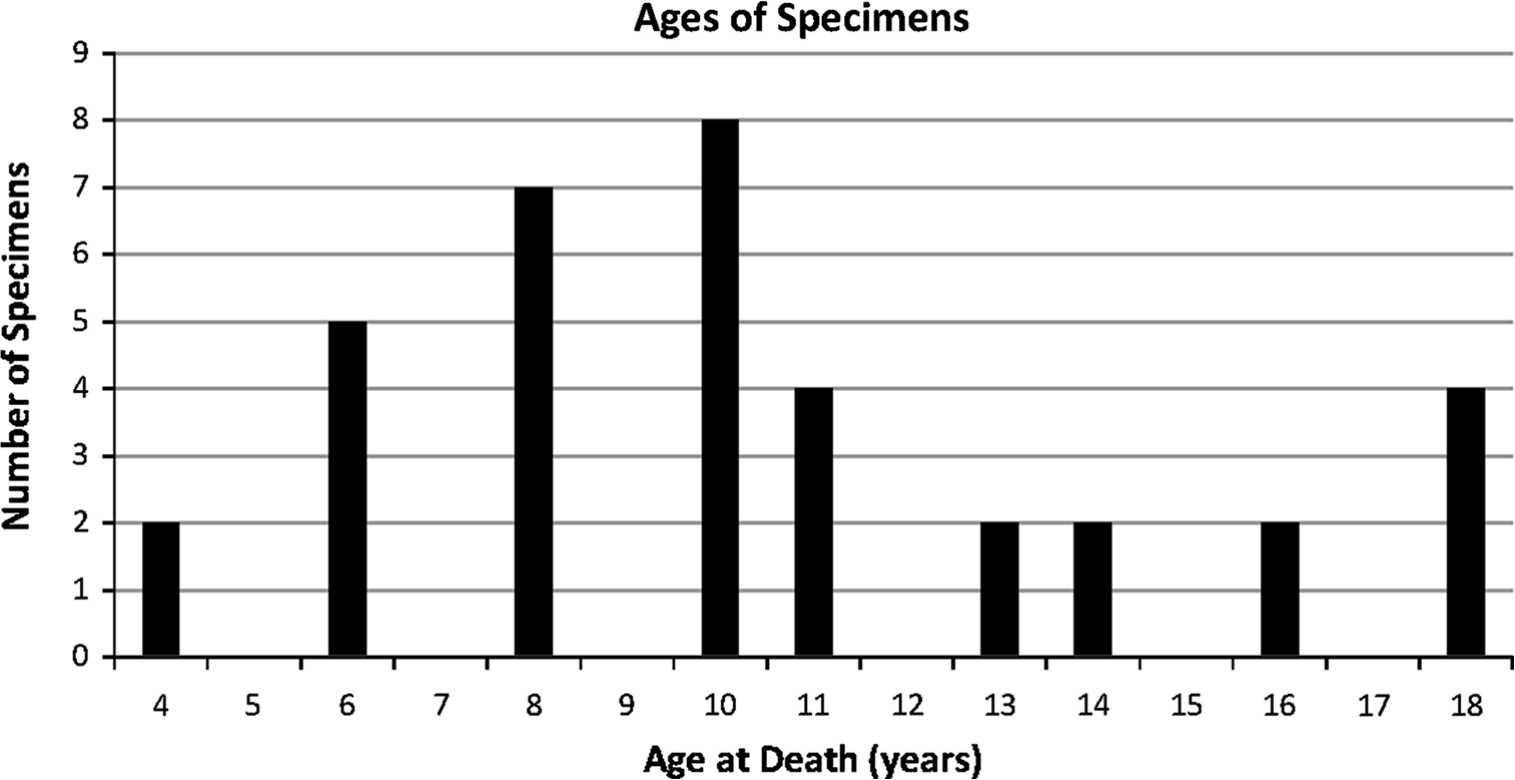
Ages at the time of death of the specimens used

The specimens studied ranged in age from 3 to 18 years. Figure 3 shows the age demographics of the study specimens.

**Fig. 4:**
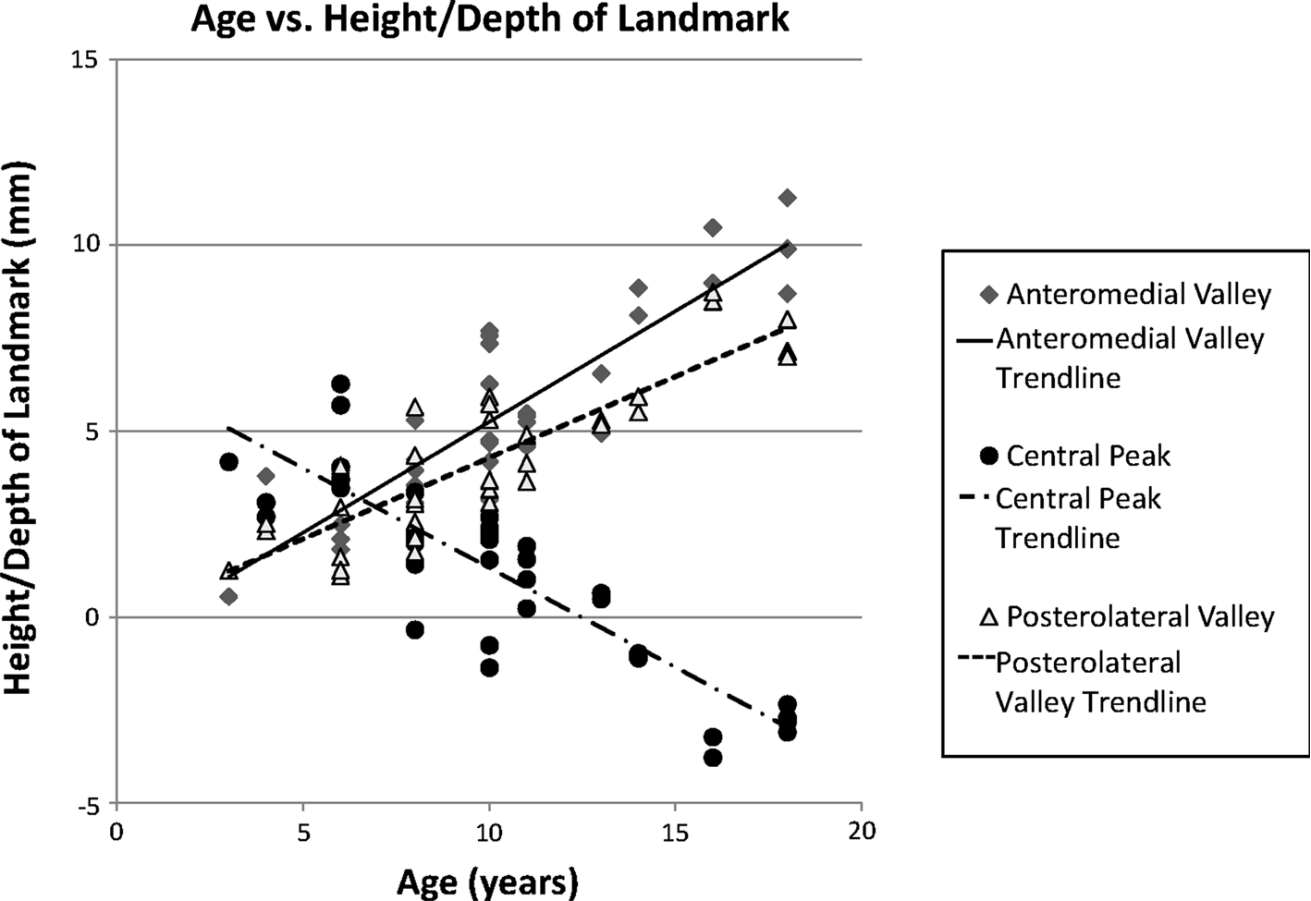
Age plotted versus height/depth of landmark, demonstrating increasing depth of the anteromedial and posterolateral valleys and decreasing height of the central peak, secondary to the increase in peripheral physeal cupping with age

The previously described central peak, anteromedial valley, and anterolateral valleys were observed on all specimens. The measurements on each radiograph view with and without markers were averaged for each landmark. As shown in Fig. 4, with increasing age, there was a corresponding relative increase in the depths of the anteromedial (*r*^2^ = 0.80) and posterolateral valleys (*r*^2^ = 0.74). Due to the increased cupping at the periphery of the physis and its effect on the physeal plate line, the central peaks became less prominent with age (*r*^2^ = 0.75).

**Fig. 5:**
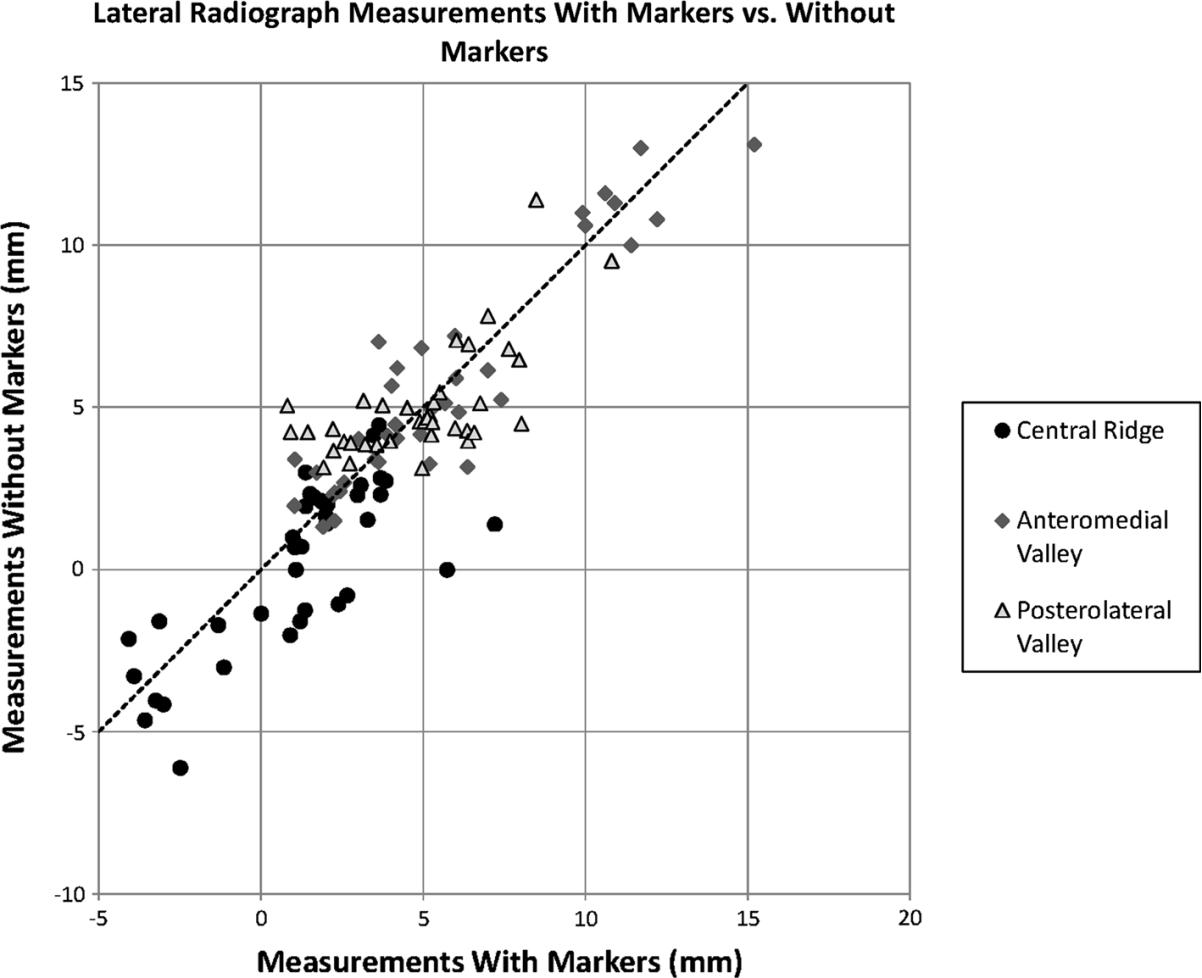
Comparison of lateral radiograph measurements with markers versus without markers. The plots demonstrate better correlation with the anteromedial valley versus the central peak and posterolateral valley

**Fig. 6:**
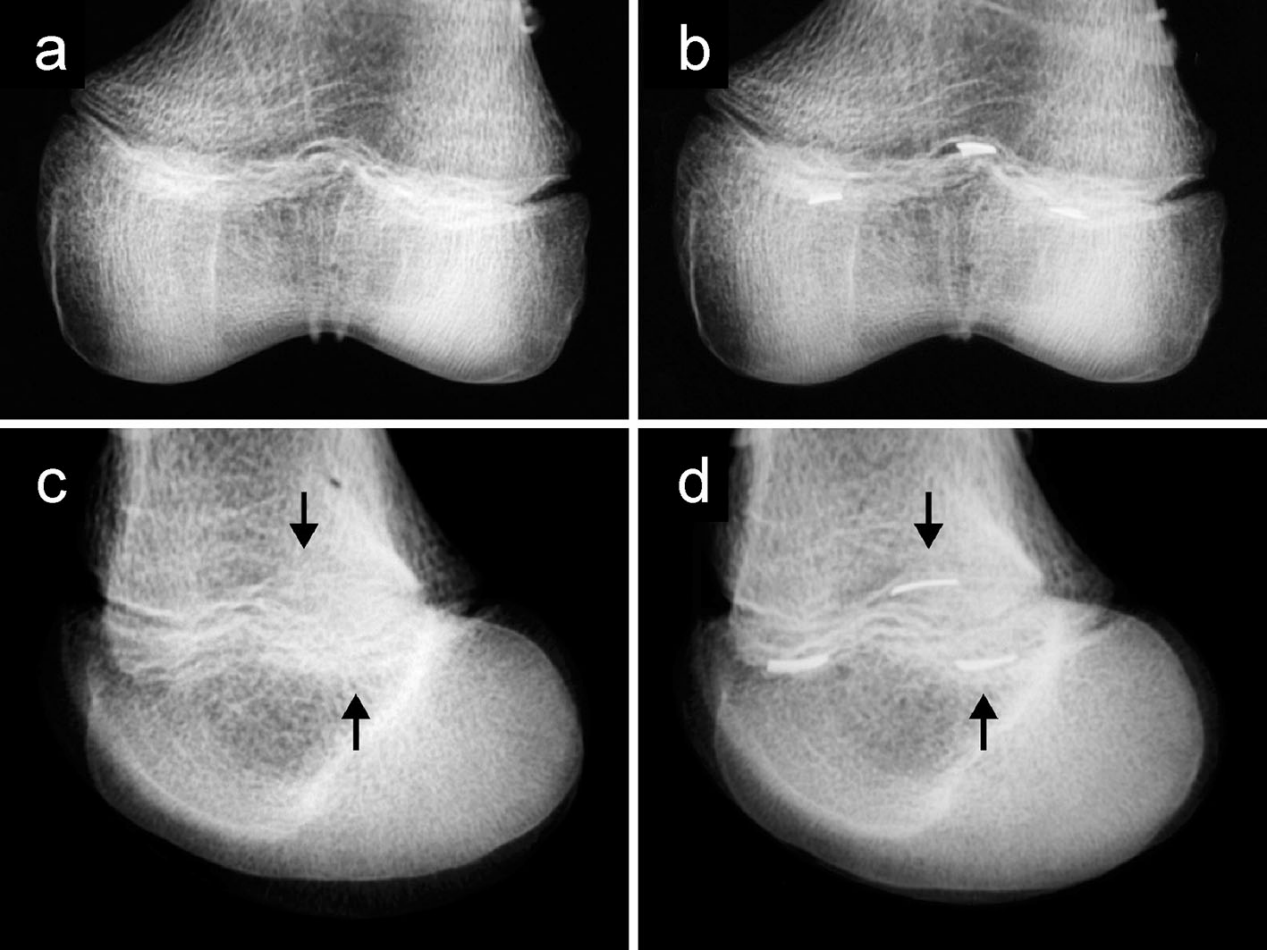
Example of visibility of landmarks on AP versus lateral view radiographs. Cadaveric distal femur of a 10-year-old male. **a** AP radiograph without markers, undulations easily visible. **b** AP radiograph with markers. **c** Lateral radiograph without markers, difficult to visualize posterolateral valley and central peak, marked by *arrows*. **d** Lateral radiograph with markers

We next compared the measurements obtained from radiographs of the specimens without markers to the radiographs taken with markers in place. On the AP views, the correlations between radiographs taken with and without markers were high for all the landmarks (ICC = 0.92, 0.92, 0.91). However, when we analyzed the measurements obtained on lateral view radiographs (Fig. 5), we found that the correlation between marked and unmarked specimens was reasonably high for the anteromedial valley (ICC = 0.94) and the central peak (ICC = 0.84), but lower for the posterolateral valley (ICC = 0.36). Figure 6 demonstrates the relative difficulty of identifying landmarks on the lateral view compared to the AP view, especially for the posterolateral valley.

We then compared the measurements obtained on the AP view radiographs to the measurements from the lateral radiographs. On the images without markers, the correlation was not high for any landmark, but the correlation for the posterolateral valley (ICC = 0.28) was considerably lower than the correlations for the central peak (ICC = 0.62) and the anteromedial valley (ICC = 0.57).

The interobserver ICC for each landmark on the radiographs with tape were excellent, ranging from 0.82 for the lateral radiographs of the posterolateral valley to 0.98 for the AP radiographs of the central peak. For the samples without tape, the range was from 0.663 for the lateral radiographs of the posterolateral valley to 0.98 for the AP radiographs of the central peak.

The intraobserver ICC for each landmark on the radiographs with tape were also good, ranging from 0.61 for the lateral radiographs of the posterolateral valley to 0.99 for the AP radiographs of the central peak. For the samples without tape, the range was from 0.76 for the lateral radiographs of the posterolateral valley to 0.98 for the AP radiographs of the central peak.

## Discussion

Iatrogenic damage to the distal femoral physis can lead to growth arrest and subsequent limb deformity. This has been directly shown in animal models [1–4, 10], as well as in a retrospective study of children who sustained inadvertent damage to the distal femoral physis during implant placement for fracture fixation [5].

A previous study of distal femoral epiphysis specimens found that the three major undulations in the distal femoral physis were the central peak, the anteromedial valley, and the posterolateral valley [6]. That study found that, with increasing age, the depths of the anteromedial and posterolateral valleys increased, reflecting the peripheral cupping effect of the physis. At the same time, the prominence of the central ridge over the physeal line decreased with increased age. Our radiographic results confirmed this previous direct anatomic study.

Although radiographs are routinely used during implant placement near the physis, the reliability of their ability to visualize these major undulations of the distal femoral physis had not been investigated until this study. Since our specimens allowed direct visualization of the undulations, our radiodense markers mapped exactly where the undulations were located on the specimens’ physes. These allowed comparison to unmarked radiographs on images taken in the same orientations that would be used in the operating room. To compare the measurements on unmarked and marked images and between different views, we used ICCs, since they are the appropriate statistical test to describe similarities between quantitative measurements of the same variable.

This protocol has not been utilized before, since we are the first group, to our knowledge, to have both access to an osteological collection this large and to explore this particular issue. The interobserver and intraobserver reliability using our methods ranged from good to excellent. The interobserver reliability for the specimens without radiodense tape, although still good, were likely not as high due to the fact that the undulations are difficult to visualize without the markers in place.

Our study found that, although AP view radiographs without markers led to measurements that corresponded well to the radiographs with markers in place, lateral view radiographs were less reliable, especially at visualizing the posterolateral valley and the central peak. The central peak was also concerning in that the measurements without markers were often lower than the measurements with markers on the lateral view (Fig. 4), suggesting that the tendency is to underestimate the size of this structure on radiographs.

The correlation between measurements taken on the AP view and the lateral view was not high for any of the landmarks. The low values of the correlation coefficients may be a result of some inconsistency between the physeal lines drawn on the AP radiographs versus the lines drawn on the lateral radiographs. Despite this, there was lower correlation in the posterolateral valley, supporting concern that this valley may be particularly difficult to appreciate on the lateral view.

The unreliability of radiographs to visualize the posterolateral valley of the physis may be of significant clinical importance, due to its peripheral location. As early as 1956, Ford and Key noted, in their study of experimental trauma to the distal femoral epiphyses of rabbits, that damage to the central portion of the physis can cause growth interference, but that damage to the periphery of the physes additionally caused growth deformity [1]. Seil et al. found similar results when they eccentrically drilled across the distal femoral physes of sheep and the animals developed femoral deformities [3]. The same animals underwent transphyseal drilling of their tibias as well, but since the drilling was done in the central portion of the proximal tibia physes, their tibias did not develop deformities.

The findings from this study are applicable to any procedure that requires hardware placement in close proximity to the distal femoral physis. The technique for the placement of plates for hemiepiphysiodesis requires adequate visualization of the physis in order to center the plate appropriately [11]. Based on the locations of the physeal undulations, placement of the distal screw in a plate too proximal and anterior on the medial side of the distal femoral epiphysis risks damage to the anteromedial valley. Analogously, a similar too proximal and posterior placement on the lateral distal femoral epiphysis risks damage to the posterolateral valley. Our findings suggest that lateral view X-rays may be unreliable, but that AP views may be used with more certainty. Another increasingly popular procedure involving implant placement in the distal femur is the MPFL reconstruction. The femoral attachment of the MPFL is located in close proximity to the medial distal femoral physis [14]. When drilling to place a fixation device to secure the femoral attachment of the graft, there is risk of accidental damage to the physis. Our study results suggest that, on a lateral view, particular scrutiny must be used to ensure that the distal femoral tunnel is not going to damage the physis. Similarly, an all epiphyseal ACL reconstruction or partial transphyseal ACL reconstruction that aims to spare the distal femoral epiphysis are other indications that require drilling near the distal femoral physis using fluoroscopy as a guide and which would benefit from our data [8, 9]. Recently, a case series using magnetic resonance imaging (MRI) to assess physeal damage after all epiphyseal versus partial transphyseal (sparing of the distal femoral physis, but violation of the proximal tibial physis) showed minimal distal femoral physeal damage and confirmed the safety of this technique when done properly [15].

This study is limited in that we used skeletal specimens that were devoid of soft tissue and cartilage. Since the physis itself was not present, we used the contact surface between the epiphysis and metaphysis as a surrogate, which seemed appropriate, given the obvious and well-preserved interdigitation between the epiphysis and the metaphysis pieces of each specimen. Positioning of each specimen to match the standard AP and lateral radiographs was challenging. Several of the radiograph images were repeated until appropriate images were obtained. Our sample size was also limited by the number of well-preserved immature femora in the bone collection, although we were able to study a diverse age range of specimens.

Although it is recognized that, in the typical operating room, C-arm fluoroscopy would be used when operating near the physis, the radiographs used in this study serve as a reasonable alternative for evaluating the visualization of the undulations. The specimens used are from a historic well-preserved collection, and the risk of damage to the delicate samples during transport is too high to justify taking the specimens out of the collection to use a C-arm unit. The use of plain radiographs may have actually made the landmarks easier to detect, since conventional radiographs have been shown to be superior to fluoroscopy in the detection of bony detail, such as pedicles and endplates [16]. Furthermore, although C-arm fluoroscopy would allow more views, the AP and lateral views are the standard views used intraoperatively to assess hardware position [17].

In summary, this study shows that, although radiographs generally visualize the undulations of the distal femoral physis well, caution should be taken when using lateral radiographs. Certain landmarks, particularly the posterolateral valley, are not reliably visualized on a lateral radiograph. We recommend that, particularly when operating near the posterolateral portion of the pediatric distal femur, fluoroscopic images in both views should be carefully interpreted and a larger distance from the growth plate be used to reduce the risk of inadvertently damaging the physis.
